# A Dataset of Pulmonary Lesions With Multiple-Level Attributes and Fine Contours

**DOI:** 10.3389/fdgth.2020.609349

**Published:** 2021-02-17

**Authors:** Ping Li, Xiangwen Kong, Johann Li, Guangming Zhu, Xiaoyuan Lu, Peiyi Shen, Syed Afaq Ali Shah, Mohammed Bennamoun, Tao Hua

**Affiliations:** ^1^Shanghai BNC, Shanghai, China; ^2^Embedded Technology & Vision Processing Research Center, Xidian University, Xi'an, China; ^3^College of Science, Health, Engineering and Education, Murdoch University, Perth, WA, Australia; ^4^School of Computer Science and Software Engineering, The University of Western Australia, Perth, WA, Australia; ^5^Pet Center, Huashan Hospital, Fudan University, Shanghai, China

**Keywords:** deep learning, radiology, pulmonary dataset, classification, attention

## Abstract

Lung cancer is a life-threatening disease and its diagnosis is of great significance. Data scarcity and unavailability of datasets is a major bottleneck in lung cancer research. In this paper, we introduce a dataset of pulmonary lesions for designing the computer-aided diagnosis (CAD) systems. The dataset has fine contour annotations and nine attribute annotations. We define the structure of the dataset in detail, and then discuss the relationship of the attributes and pathology, and the correlation between the nine attributes with the chi-square test. To demonstrate the contribution of our dataset to computer-aided system design, we define four tasks that can be developed using our dataset. Then, we use our dataset to model multi-attribute classification tasks. We discuss the performance in 2D, 2.5D, and 3D input modes of the classification model. To improve performance, we introduce two attention mechanisms and verify the principles of the attention mechanisms through visualization. Experimental results show the relationship between different models and different levels of attributes.

## 1. Introduction

Lung cancer is caused by tumors which leads to the fastest increase in morbidity and mortality. It has a significant negative impact on the health of subjects. Therefore, the early diagnosis of lung lesions is of great significance for the treatment of lung cancer.

The early form of lung cancer is categorized as pulmonary nodules, which are clinically examined using computed tomography (CT). The characteristics of pulmonary nodules in CT images are diverse, which results in a large workload for radiologists to diagnosis the disease and leads to the subjective assessment of features. Therefore, accurate and quantitative analysis of the appearance characteristics of lung nodules is very essential for doctors to determine whether the nodules will grow into malignant tumors.

In recent years, with the development of deep learning technology (1), lung nodule diagnosis has made unprecedented progress in detection (2–7), segmentation (8–11), classification (2, 6, 12–15), and registration (16, 17) tasks. In order to improve the performance of the model, there is a great need of large datasets and accurate annotation of pulmonary lesions.

There are many publicly available datasets of pulmonary nodules. However, there are some shortcomings in the existing datasets, and the diversity of lesions cannot be balanced in these datasets. For example, LIDC/IDRI (18) has rich attributes, however, it only marks nodules, and the prediction of other pulmonary diseases cannot be performed.

In this paper, we propose a dataset of lung lesions that could help the development of a pulmonary computer-aided diagnosis system. Our dataset is multi-centered, data-diversified, and informative. The proposed dataset is rich in lesion types and covers most of the signs of lung lesions. The lesions of the dataset are labeled with contours and attribute annotations by experienced radiologists using a professional tool. The attribute annotations are composed of nine attributes that are most useful for pathological assessment. In order to make the selected attributes hierarchical, we have selected multi-level attributes:

- **Low-level attributes**: Margin, spiculation, etc, which can be judged basically by the local features of the lesion;
- **Middle-level attributes**: Pleural indentation, vessel convergence, etc, which need to be judged by the relationship with the surrounding tissue around the lesion or cavity and calcification, which need to be judged by the relationship between local features and global features of the lesion;
- **High-level attributes**: The type and the location of the lesion, which requires to be judged by the abstract features of the entire lesion.

In order to describe the proposed dataset clearly, we first count the characteristics of our dataset, define the data storage format and data annotation rules for our dataset. We then propose the contours annotation format. We also focus on the correlation between the attributes of the lesions. In order to study the relationship between multiple attributes, we calculated the probability of a total of 27 categories of 9 different attributes using the chi-square test and conditional probability, and infer the correlation with the attributes by probability.

In order to illustrate the practical significance of our dataset, we discuss several applications that could be studied using our dataset, and then select the attribute classification for further study. First, we model the attribute classification and then explored the performance of the 2D, 2.5D, and 3D input modes on the accuracy of the model. Through experiments, we demonstrate that there is implicit competition between multiple attributes, we, therefore, use two attention mechanisms to filter different feature activations for different attributes. Our experiments show that the attention mechanisms have different effects on attribute classification.

## 2. Related Work

In this section, we briefly discuss the existing datasets of lung nodules and the relevant classification methods.

### 2.1. Lung Nodule Datasets

#### 2.1.1. LUNA16 Dataset

The LUNA16 (4) dataset was designed for the Open Pulmonary Nod Challenge, which screened 888 CT volumes from a large dataset LIDC/IDRI as challenge data. Their slice thickness is within 2.5 mm and the nodule size is greater than 3 mm, which was annotated by more than 3 experimental doctors using tow-phase annotation. The detection annotations of a nodule in LUNA16 use the center coordinates and diameter of the inscribed circle of the nodule. In contrast, we use the gravity center coordinates as the center coordinates of the nodule and the longer geometric moment as the diameter to generate the world coordinates. For small round nodules, the two datasets are not much different, but the need is to detect large lesions with irregular shapes and our proposed approach achieves better results for large lesion detection.

#### 2.1.2. LIDC/IDRI Dataset

The LIDC/IDRI (18) dataset labels each nodule with a contour and nine attributes. Besides the benign and malignant nodules, the other eight attributes are all the appearance attributes of the nodules. In contrast, in our dataset, two of the attributes are the basic attributes of the lesion, five are appearance attributes, and two have relationships with the tissue surrounding the lesion in context. These attributes are richer and can better represent a lesion.

#### 2.1.3. LISS Database

The LISS (19) database has 271 CT volumes, including 677 abnormal regions. These abnormal regions are divided into nine categories, which are called common CT imaging signs of lung disease (CISLs). In other words, there is only one CISLs label for each abnormal region. Although it can better help medical scholars learn a certain type of disease (12), it is not very good for CAD system development, because it cannot capture the relationship between disease signs.

#### 2.1.4. ILD Database

The ILD (20) database has 108 image series with more than 1946 ROIs. This dataset is a multimedia collection of cases of interstitial lung disease (ILDs). These ROIs are divided into 13 categories, which are lung tissue patterns from histological diagnoses of ILDs. The lesions in the ILD dataset are large, and the annotations are all high-level attributes. The dataset does not focus on a certain nodule, but on the pathology presented by a piece of tissue.

### 2.2. Lung Nodule Classification

The classification of lung nodules based on deep learning can be divided into two types of methods: one is to judge the benign and malignant lung nodules. Some methods directly predict the benign and malignant nodules by CT images, and other methods use different attributes of the nodules as the auxiliary basis to judge the benign and malignant nodules, such as (21–23). The other type of method has classified the disease, such as DeepLung (2) or LISCs classification (12). Dey et al. (21) have built a network that produces multiple outputs from multi-scale features to judge the benign and malignant nodules. Nibali et al. (22) has made a three-column configuration to fuse the features generated from three axes. Song et al. (14, 23) proposed methods that split the whole image into patches and predict the lesions. In contrast, Gao et al. (13) have used the whole image for classification. With the development of computationally efficient computers, the 3D models such as (24) has achieved an impressive performance in nodule classification. He (12) proposed a method to generate images for data augmentation, which achieved a good improvement in performance. Zhu et al. (2) detected the position of the nodules first, then cropped the sent the nodules before feeding it into a classification model to predict one of nine attributes.

Multi-attribute classification is a problem to classify multiple targets using one model. There are currently two approaches to solve this problem. The first is to regard it as a classification task with a fixed number of categories, and solve attribute correlation in one model by using multiple branches to decompose the relationship between multiple targets onto each branch. The second is to treat it as a multi-label classification task, with the positive attribute as the label of the lesion, then each lesion has a floating number of labels, and the labels are decoupled using different methods. In this paper, we use the first method to classify different attributes in a model using a fixed number of branches, and use two attention mechanisms to help decouple the correlation among the attributes.

## 3. Lung Lesion Dataset

**Figure 1:**
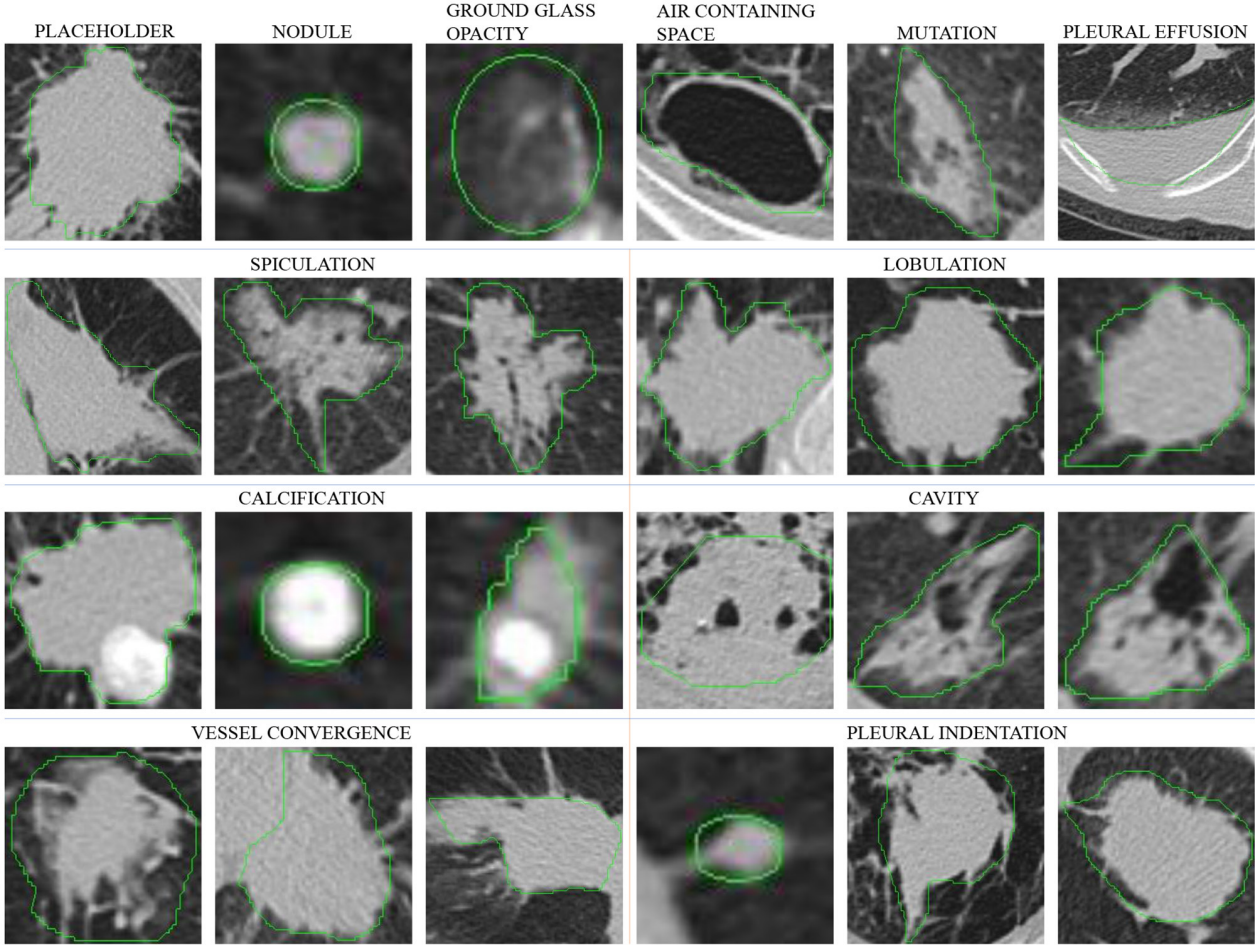
Lesions in our dataset. Except for some small nodules, which are marked with a circle, such as the second image in the first row, other lesions are marked by a very close contour. The six images in the first row are different types of lesions, and in the second to fourth rows, each set of three images are spiculation, lobulation, calcification, cavity, vessel convergence, and pleural indentation.

In this section, we provide a description of our dataset. CT data were collected from four hospitals. The body parts examined are mainly the chest and abdomen. Among them, the chest CT was mostly thin (less than 3 mm), and the abdomen CT was mostly thick (greater than or equal to 5 mm). Figure 1 shows examples of lesions in our dataset. As shown in Figure 1, except for some small nodules, which are marked with circles, such as the second image in the first row, other lesions are marked by a very close contour.

Table 1 shows the parameter comparison of our dataset with several other public datasets. Same with LUNA16, our dataset annotates lesion with contour, which is shown in Figure 1. Compared with box and polygon, contour annotation has more generalization ability to different tasks, such as location, detection, and segmentation. At the same time, though the number of scans in our dataset is not the largest, the number of lesion annotations and the range of lesion size in our dataset are. These annotations support more robust models. Moreover, the thickness of the slices of our datasets is relatively uniform, especially compared to LUNA 16. It reduces unnecessary processing of the data and makes it easier to use.

**Table 1:** The statistical result of comparing our dataset parameters with other datasets.

**Dataset**	**Annotation**	**Lesion attributes**	**Multiple categories**	**Scans**	**Lesion amount**	**Lesion size (mm)**	**Slice thickness (mm)**	**Pixel spacing (mm)**
LUNA16	contour	9	✓	888	1,186	3.25–32.27	0.45–2.50	0.461–0.976
LISS (2D)	Box	9	×	252	511	–	5.0	0.42–1.00
LISS (2D)	Box	9	×	19	166	–	1, 1.25	0.60–0.87
ILD	Polygon	13	×	108	1,946	–	1.00–2.00	0.40–1.00
Ours	Contour	9	✓	694	5,113	0.83–191.32	1.00–2.00	0.176–0.977

### 3.1. File Storage and Annotation Format

The raw data obtained from the hospital contains some sensitive information of subjects, and the data collected from different hospitals are stored in different ways, making the data difficult to use directly for analysis. Therefore, we first desensitize the data by removing subjects' sensitive information and retain only the necessary information, such as weight. Then, we store the CT volumes and annotation files as described below.

We define the directory structure to store files as follows:


ct_type/hospital/year/month/day/subject_id/series_id.


The directory with series_id SE01 stores the CT data with DICOM format, and the directory with series_id SE01_01_0*n* stores the contour annotation file aid_loc .anno, where *n* is the identification number of the doctor who annotated the scans; aid is the number of the annotation in the CT for correspondence with the attribute information; loc is the slice number in the CT volume, and the description in the DICOM file is SliceLocation (0020, 1041). An anno file represents an annotation. Each anno file has a different aid, but two anno files can have the same loc, indicating that the two annotations are in the same slice. It uses a dictionary to store the annotation information we need to use in the CAD tasks. The keywords of the anno format are SeriesID, NoduleSerialNumber, InstanceNumber, Origin, Dimension, Spacing, Coords, XMin, XMax, YMin, YMax. Among them, SeriesID is a unique number of a DICOM volume which described as SeriesInstanceUID (0020, 000E), NoduleSerialNumber and InstanceNumber are aid and loc, respectively as mentioned above, Origin, Dimension, Spacing are the information from DICOM volume, Coords is the contour coordinate of this annotation, and its value is relative to the size of this slice. (XMin, YMin), (XMax, YMax) are the coordinates of the lower left and upper right corners of the bounding box of this annotation.

The CT volumes in our dataset contain lesions, while those without lesions have been removed by manually screening of RIS reports. For repeated subject numbers, such as two volumes of one subject, we map one of them to a new subject number and retain the correspondence to restore the original number.

### 3.2. Two-Phase Annotation Process

We use a two-phase annotation process to label the lesions. We label the contours of the lesions in the first phase, then label the attributes of the lesions in the second phase.

#### 3.2.1. Contour Annotation Criterion

The contours are marked by experienced radiologists. In order to save the doctor's time and to increase the density of the lesion, we first manually screen the RIS report, retain the CT volume with the lesion in the description, and remove the volume without the lesion from the dataset. In order to standardize the process of marking the lesions, we have prescribed a rule for marking lesions with the doctor as follows:

- Mark all visible lesions;
- If the lesion is too small to draw the contour, circle the lesion with a circle tool;
- If the lesion is larger than one slice, mark the lesion every three consecutive slices;
- Draw a contour as close as possible to the edge of a lesion.

After the marking process, we perform a secondary screening to remove the annotations which are too discontinuous to be processed as contours. Then, we convert the annotations into anno format and mark lesion numbers. In this way, the contour annotations and the attribute annotations correspond with respective file names.

#### 3.2.2. Attribute Annotation Criterion

After discussed with the doctor, we selected nine attributes that are commonly used in clinical diagnosis as attribute annotations for the dataset. A detailed description of these attributes will be provided in section 5.2. Each lesion is independently labeled by a doctor, and we record the doctor's number for each lesion that can be used to identify the doctor if an error is discovered in the annotation.

In order to simplify the labeling of attributes, we implement an attribute labeling tool to collect and manage labels. We associate the slice of the contour with the lesion number so that it is convenient to label the attributes with the corresponding slice. When the attribute information is marked, the corresponding subject number and label number are recorded to correspond to the contour number. It should be noted that the contour annotation and the attribute annotation are not one-to-one matched. Some problematic contour annotations are filtered out in the previous step, and no attribute annotation is performed. Finally, we only select lesions with both contour and attribute annotations into the dataset. The number of attributes is reported in Table 1. As can be noted, the categories of some attributes are very unbalanced. This brings great challenges to the performance of our attribute classification algorithm.

## 4. Attributes and Pathology

We initially selected 15 attributes that are commonly used in clinical diagnosis, and then selected 9 attributes for our dataset based on their importance. The number of categories of these attributes is not balanced and the distributions are not independent. Here we briefly describe the importance of these attributes in clinical diagnosis and then discuss the correlation between attributes from the statistics point of view.

### 4.1. Attributes Description

Among the 9 attributes we selected, besides the basic attribute, lesion type, and lesion location, there are vessel convergence and pleural indentation which represent the relationship between the lesion and the surrounding tissue. On the other hand, margin, calcification, lobulation, spiculation, cavity represent the apparent features of the lesion. The description of the significance of these nine attributes is as follows.

#### 4.1.1. Lesion Type

The first row of Figure 1 shows six different lesion types. For the lesion type, we choose *placeholder, nodule, ground glass opacity, air containing space, mutation*, and *pleural effusion*. The difference between placeholders and nodules is that the lesions with a diameter of less than 30 mm are nodules, and those larger than 30 mm are placeholders. Except for the difference in size, the other attributes of the two lesion types are roughly similar. The air containing space is different from the cavity in pathology. The air containing space (Figure 1, the fifth image in the first row) is a pathological enlargement of the physiological cavity in the lung, while the cavities (Figure 1, the last three images in the third row) often appears in nodules or placeholders. In the air containing space lesions, the wall of the lesion is thinner and more uniform, mostly occurring in the subpleural area, and the size varies greatly. This means that the location of the air containing space is fixed and there are no apparent attributes such as spiculation and lobulation.

#### 4.1.2. Lesion Location

The location of the nodule is represented by five categories of lobes, including the *right upper* lobe, the *right middle* lobe, the *right lower* lobe, the *left upper* lobe, and the *left lower* lobe. Statistics show that the occurrence of lesions has little relationship with the location. The lesion location is only a basic attribute of the lesion, and it cannot be used as a basis for judging its pathological nature. Some lesions are large and span multiple lung lobes, so we mark them as 0, and do not include it in the five categories above.

#### 4.1.3. Margin

The margin attribute describes whether the outer boundary of a nodule is clear. We defined two main categories for this attribute: *clear* and *unclear* margin. Though the margin of a benign mass is often smooth, while that of a malignant mass is often unclear, inflammation may also cause an unclear margin of placeholder. Therefore, it cannot be used as the sole basis for judging benign and malignant lesion, and needs to be judged in combination with other attributes.

#### 4.1.4. Calcification

The calcification attribute describes lesions whose density is significantly higher than other soft tissues in the mediastinal window, usually with CT values above 100 Hu. The first three images in the third row of Figure 1 show lesions of calcification. The white region in the images represents calcification. Calcification is a pathologically metamorphic lesion, which is more common in the healing stage of ductal tuberculosis lesions in the lung tissue or lymph nodes; calcification can also occur in tumor tissues or cyst walls. Usually, the greater the proportion of calcification in the lesion, the greater the likelihood of its being benign. Based on this, we classify the calcification attributes into three categories: *no, partial*, and *total* calcification.

#### 4.1.5. Lobulation

The lobulation attribute indicates that the nodule or mass grows at different speeds in various directions or is blocked by the surrounding structure. The contours may have a plurality of arcuate protrusions, and the curved phases are concave cuts to form a lobulated shape. The last three images in the second row of Figure 1 show the lesions of lobulation. We can clearly see the convex part of the masses. We simply define two categories for this attribute: *with* and *without* lobulation.

#### 4.1.6. Spiculation

The spiculation attribute is characterized by a radial, unbranched, straight, and strong thin line shadow extending from the edge of the nodule to the periphery, and the proximal end of the shadow is slightly thicker. The first three images in the second row of Figure 1 show lesions of spiculation. As shown in Figure 1, the burrs of the lesion are often not circled in the scope of annotation. The spiculation is not connected to the pleura, and distinct from the pleural depression. We classify the spiculation attributes into *no, short* and *long* spiculation; 5 mm burrs are called short spiculation, and larger than 5 mm burrs are called long spiculation. The pathological basis of the burr is the fiber band in which the tumor cells infiltrate into the adjacent bronchial sheath and local lymphatic vessels, or the tumor promotes connective tissue formation. Benign nodular inflammatory pseudotumor, tuberculoma can also be seen burrs, but longer, softer, more often formed by hyperplastic fibrous connective tissue. The possibility of lung cancer should be considered when there is a burr in solitary lung nodules.

#### 4.1.7. Cavity

The cancerous cavities are mostly located in the anterior segment of the upper lobe and the basal segment of the lower lobe. Most of the cavities larger than 3 cm in diameter are tumors. Most cancerous cavities present an irregular or lobulated outer edge and irregular inner edge. Those with a wall thinner than 4 mm are mostly benign lesions, and those thicker than 15 mm are mostly malignant lesions. The last three images in the third row of Figure 1 show the lesions of a cavity. We simply defined two categories for this attribute: *with* and *without* cavity.

#### 4.1.8. Vessel Convergence

The vessel convergence attribute appears on the slices as one or more vessels around the pulmonary nodule that touch with, cut or pass through the placeholder at its edge. The appearance of vessel convergence is related to the size of the placeholder or nodule. The lesions less than 1 cm in diameter have fewer vessel convergence signs. The first three images in the last row of Figure 1 shows the lesions of vessel convergence. Images of the cavities and vessel convergence are similar, because the blood vessels look like cavities when they are transacted. A multi-vessel-directed lesion presents vessel convergence, which leads to a higher chance of malignancy. In particular, the phenomenon that one blood vessel leads to a nodule or tumor is not only seen in malignant nodules, but also in benign lesions such as tuberculosis, inflammatory pseudotumor, or hamartoma. We simply defined two categories for this attribute: *with* and *without* vessel convergence.

#### 4.1.9. Pleural Indentation

The typical pleural indentation shows a small triangular shadow or a small trumpet shadow on the visceral surface of the visceral pleura. The bottom of the triangle is on the inside of the chest wall, the tip points on the nodule, and the nodule and the triangle shadow can be connected by a linear shadow. The last three images in the last row of Figure 1 shows the lesions of pleural indentation. Peripheral lesions of the pleural indentation are often accompanied by other imaging signs. The pathological basis and imaging manifestations of pleural indentation in benign and malignant lesions are different. We simply define two categories for this attribute: *with* and *without* pleural indentation.

### 4.2. Correlation Between Attributes

In order to evaluate the correlation between attributes, we used the chi-square test. We assume that if the two attributes are independent of each other, their data distribution should not affect each other, which means that the proportional relationship between the categories of one attribute is the same under each category of the other attribute. If the chi-square test value calculated by the two attributes is greater than the statistical significance, there is a correlation between the two attributes. The approximate calculation equation for the chi-square test statistic is as follows:


(1) $$\begin{array}{l} \chi^{2}=\sum \frac{{\left(f_{0}-f_{e}\right)}^{2}}{f_{e}} \end{array}$$

where *f*_0_ is the actual number of observations and *f*_*e*_ is the expected number of times. The larger the value of *f*_*e*_, the Equation (1) approximately obeys the chi-square distribution. To simplify the calculation of the chi-square test, we used a variant of Equation (1):


(2) $$\begin{array}{l} \chi^{2}=\sum \frac{{\left(f_{xy}-\frac{f_{x}f_{y}}{N}\right)}^{2}}{\frac{f_{x}f_{y}}{N}}=N\left(\overset{R}{\underset{x=1}{\sum }}\overset{C}{\underset{y=1}{\sum }}\frac{f_{xy}^{2}}{f_{x}f_{y}}-1\right) \end{array}$$

where *f*_*x*_ and *f*_*y*_ represent the number of samples of the categories of two different attributes *x* and *y*, respectively, *R* and *C* are the number of categories of *f*_*x*_ and *f*_*y*_, and the total number of attributes is *N*. The degree of freedom *df* of the independence test is calculated as follows:


(3) $$\begin{array}{l} df=R\times C-R-C-1=\left(R-1\right)\left(C-1\right) \end{array}$$

**Figure 2:**
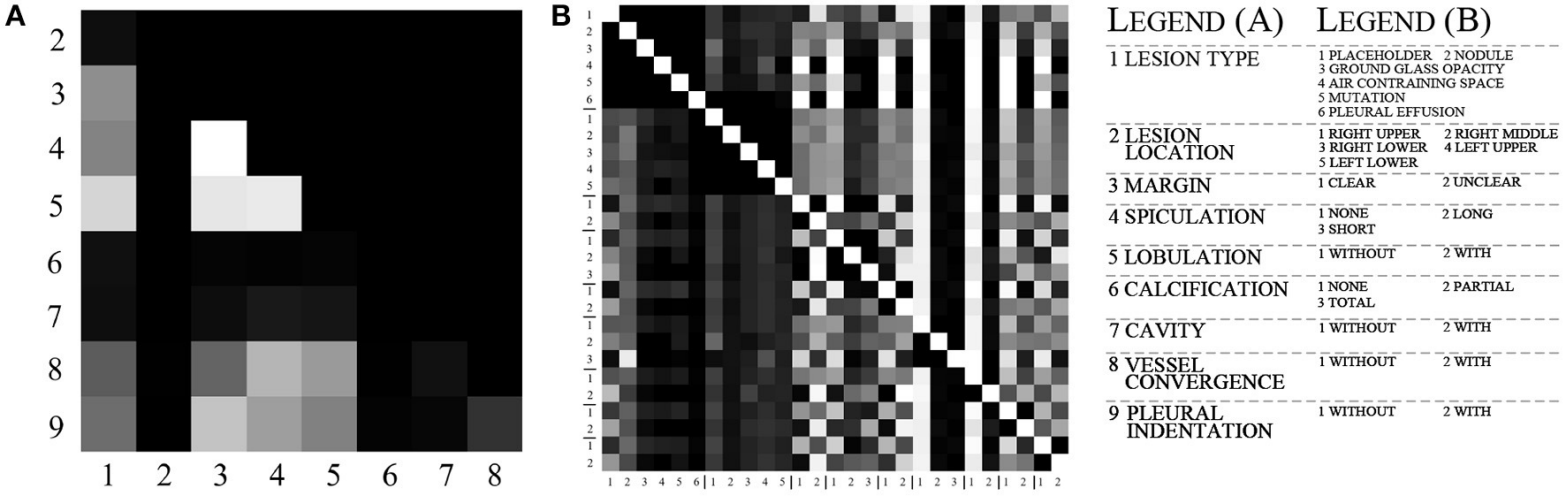
The visualization of chi-test and conditional probabilities. **(A)** is the visualization of the chi-test result. **(B)** is the visualization of conditional probabilities. The brighter grid means that the attributes indicated by its row and column numbers are more relevant. For **(A)**, the meaning of labels 1–9 is listed in legend, and for **(B)**, the legend lists the meaning of label in each group.

We use the data shown in Table 2 and select a significance level of 0.05 for calculation. Figure 2A shows the result of the chi-square test. As the results show, there is a strong correlation between the three attributes of margin, speculation, and lobulation. Meanwhile, there is a strong correlation between vessel convergence and spiculation, margin, lobulation and lesion type, pleural indentation, and margin.

**Table 2:** The distribution of each attribute category used for experiments.

**Attribute**	**Categories**	**Lesions**	**Attribute**	**Categories**	**Lesions**	**Attribute**	**Categories**	**Lesions**
Lesion type	Placeholder	675	Lesion location	Right upper	496	Calcification	None	1,902
	Nodule	728		Right middle	151		Partial	62
	Ground glass opacity	220		Right lower	286		Total	50
	Air containing space	153		Left upper	374	Cavity	Without	1,924
	Mutation	208		Left lower	271		With	90
	Pleural effusion	30	Margin	Clear	887	Vessel Convergence	Without	1,461
Spiculation	None	1,198		Unclear	1,127		With	553
	Long	307	Lobulation	Without	1,015	Pleural Indentation	Without	1,222
	Short	509		With	999		With	792

To further explore the specific relationship between the various categories of attributes, we calculated the conditional probability between a total of 27 categories for all attributes. The equation for calculating the conditional probability is as follows:


(4) $$\begin{array}{l} P\left(X|Y\right)=\frac{P\left(XY\right)}{P\left(Y\right)} \end{array}$$

where *P*(*X*) and *P*(*Y*) represent the probabilities of two categories *X* and *Y*, *P*(*X*|*Y*) represents the probability of *X* to occur when *Y* is present, and *P*(*XY*) represents the probability of co-occurrence for *X* and *Y*. The value of *P*(*X*|*X*) is 1, which is represented by white color in Figure 2B. We calculated the conditional probability between each of the two categories. As shown in Figure 2B, the white color represents a probability of 1 and the black color represents a probability of 0, while the lighter gray color represents higher conditional probability values.

According to the statistical results, there is a strong correlation between different lesion types and other attributes. For the placeholder, their margins are almost unclear, the degree of lobulation is more obvious, the degree of spiculation and the degree of pleural indentation are the highest among other lesion types. The nodules, ground glass, and mutation categories have a small number of spiculation and lobulation, and more features of vessel convergence and pleural indentation. For cavity and pleural effusion, they almost have no other attributes and their margins are all clear.

The margin attribute is highly correlated with lobulation, vessel convergence, and pleural indentation. When vessel convergence and pleural indentation are present, they are often accompanied by lobulation, and the margin is not very clear. The calcification attribute is concentrated in the nodules, and the cavity is also related to the margin and lobulation.

## 5. Tasks of Dataset

Our dataset is rich in data and diverse in annotations, which means that our dataset can be used for several tasks and aid in the development of CAD systems. We recommend using our dataset for the following tasks:

(1) **Detection**: Some of the lesions in our dataset are smaller than 30 mm, which are nearly circular and suitable for lung nodules detection. This can be helpful for the initial diagnosis of lung cancer.
(2) **Segmentation**: The lesions larger than 30 mm are all marked with precise contours. These lesions are more complex in shape and are suitable for the lung lesion segmentation task. This can be helpful for volume measurement and further treatment.
(3) **Classification**: Multiple attributes of the lesion are suitable for multi-task lung disease prediction. This can be helpful to judge benign and malignant tumors.
(4) **Reconstruction**: At present, medical datasets are small, and their size is not enough for deep learning. Our dataset has various types of data, and we can use real data to train generative adversarial networks to generate synthetic data.

In this paper, we focus on exploring the correlation between attributes. We, therefore, perform multi-attribute classification and report our experimental results in section 6.

### 5.1. 2D, 2.5D, 3D Modes for Classification

In order to study the importance of the input mode for the model, we use different data dimensions for the same data and the model for classification experiments.

We use three input modes including 2D, 2.5D, and 3D. Assuming that the size of a CT volume is *H* × *W* × *C*, which corresponds to the three axes of X-Y-Z, the diameter of a lesion is *d*, the three input modes are expressed as follows:

#### 5.1.1. 2D Mode

The lesion is cut out from the grayscale slice in which it is located with a length *d* of side, and fed to a 2D network for prediction. The input size is *d* × *d* × 1. The 2D input mode can retain the lesion at the spatial structure in the X-Y direction, but the context information in the Z direction cannot be captured.

#### 5.1.2. 2.5D Mode

The grayscale image of the lesion and the five images above and below are cut out by the bounding box, and fed to the 2D network for prediction. The number of input channels is 5, and the input size is *d* × *d* × 5. Compared to the 2D input mode, the 2.5D input mode is supplemented by a fixed number of slices in the Z direction.

#### 5.1.3. 3D Mode

In the X-Y-Z direction where the lesion is located, the bounding box (*d* × *d* × *d*) is cropped and fed to the 3D network for prediction. 3D network can capture the correlation on the Z-axis of the whole lesion by convolution. Compared with 2D, the information of 2.5D is more detailed, but the amount of 3D network parameters is more than that of 2D network, which can cause the deep learning model to overfit as the size of training data is small.

**Figure 3:**
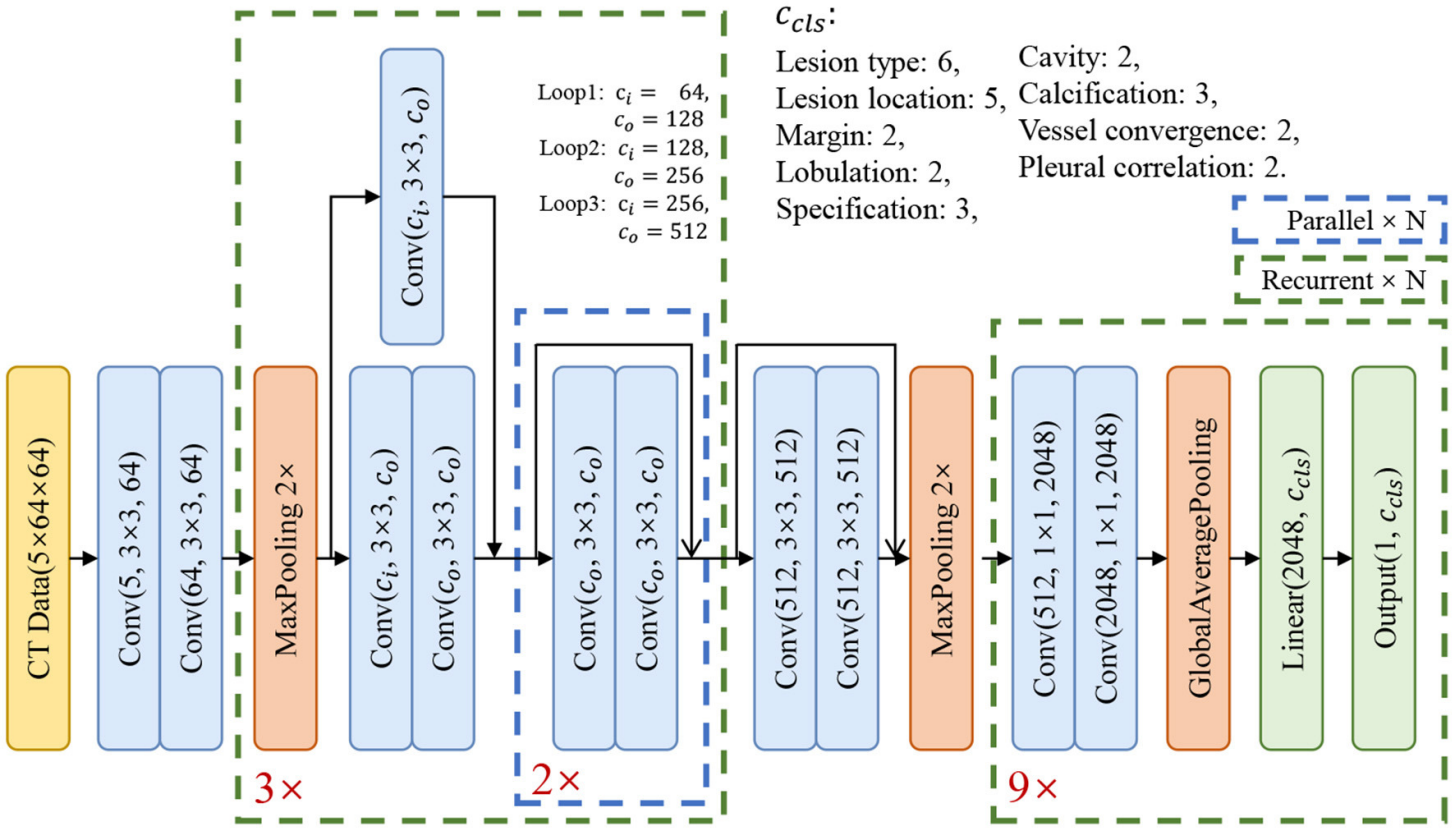
The structure of our basic classification model.

The architecture of our basic model is shown in Figure 3. In order to extract the relationship of nine attributes, we use a ResNet-based network (25) to extract the characteristics of the nodule and then use nine classification branches to predict nine attributes independently. We will explain the details and the results in section 6.1.

### 5.2. Two Attention Mechanisms

**Figure 4:**
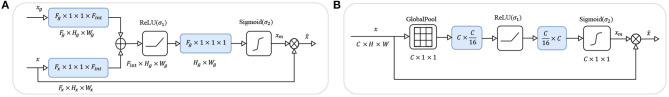
The structures of the two attention modules. In the figures above, blue boxes represent the convolutional layers, ⊕ represents the element-wise sum, and ⊗ represents the spatial-wise reweight in **(A)** and channel-wise reweight in **(B)**.

Through the experiments, we found that there is an implicit competition between multiple attributes during training. In the training phase, when the loss value is stable, the accuracy of some attributes increases while the accuracy of other attributes decreases. To solve this problem, we add an attention module in front of each attribute classifier to focus the activation on the features which are useful for classification. In this way, different input features for attributes are extracted, which could mitigate the conflict between attributes. Inspired by (26–28), we employed soft-attention and self-attention, commonly used mechanisms that compute a weight matrix used to filter noise and to focus on important features. These two attention mechanisms are described below in our model, and Figure 4 shows the structure of the two attention modules.

#### 5.2.1. Soft-Attention Module

As shown in Figure 4A, we add a soft-attention module (26) before feeding the features into the classifier to filter out shallower features with deeper features. While preserving the spatial structure, the attention module extracts a mask from the features to suppress noise which is not related to the attribute to improve accuracy.

Assuming that feature map $x\in {\mathbb{R}}^{N\times C_{x}\times H\times W}$ from the basic model is the input feature for the attention model, and feature map $x_{g}\in {\mathbb{R}}^{N\times C_{g}\times H\times W}$ is from a deeper layer as the gate, we firstly use 1 × 1 convolutional layer to get the same number of channels *C*_*g*_ for both the features, then sum the features *x* and *x*_*g*_ together and add a non-linear transform ReLU which can be formulated as σ_1_(*x*) = *max*(0, *x*). So far, the feature *x* is mixed with richer semantic information *x*_*g*_, and we use a 1 × 1 convolutional layer to fuse the channel information and retain the spatial information, and get a mask *x*_*m*_ with a value of [0, 1] through the sigmoid function which can be formulated as $\sigma_{2}\left(x\right)={\left(1+e^{-x}\right)}^{-1}$. Finally, we use the mask *x*_*m*_ to spatial-wise reweight the feature map *x* and get the output feature $\hat{x}$. After filtering by the soft-attention module, the features $\hat{x}$ are re-weighted by high-dimensional semantic information in the spatial dimension, which is more conducive to multi-attribute classification.

#### 5.2.2. Self-Attention Module

As shown in Figure 4B, we add a self-attention module (27, 28) before the features and fed to the classifier to squeeze the spatial structure of a feature map into one vector with spatial information. Then, we gather and filter the information to enhance the activation related to that attribute, and add the information to the original feature map to enhance the feature.

Assuming that feature map $x\in {\mathbb{R}}^{N\times C_{x}\times H\times W}$ is generated from the basic model as the input feature of the attention model, we use a channel squeeze and spatial excitation branch to transform *x* to extract the spatial information and reweight the origin *x* with the transform of itself. We use a global pool which can squeeze *x* to a vector $z\in {\mathbb{R}}^{N\times C_{x}\times 1\times 1}$. Then use two fully connected layers to transform the vector *z* to ẑ = *W*_1_(σ_1_(*W*_2_ · *z*)) with $W_{1}\in {\mathbb{R}}^{C\times C/16}$ and $W_{2}\in {\mathbb{R}}^{C/16\times C}$ and the activation σ_1_. We also use the non-linear function σ_2_ to transform the values to [0, 1] to get the channel mask *x*_*m*_. Finally, we use the *x*_*m*_ to channel-wise reweight the feature map *x* and get the output feature $\hat{x}$. After filtering by the self-attention module, the features $\hat{x}$ are re-weighted by the information after squeeze and excitation in the spatial dimension, which is more conducive to multi-attribute classification.

## 6. Experimental Results

In this section, we first verify that the proposed model can learn the correlation between attributes, and then empirically select the best input mode, and verify the attention mechanism on this input mode.

We used part of the data with a thickness of 1.0–2.0 mm in our experiments, which has 355 CT volumes and 2014 lesions labeled with 9 attributes in our dataset. The dataset has been split into 8:2 as the training set and validation set, with 1,847 lesions in the training set and 163 lesions in the validation set. During training, we randomly select 30% of the data for data augmentation i.e., random flip and rotation. As Table 2 shows, the number of categories in the dataset is unbalanced, which could affect the convergence of the model. We use weighted cross entropy loss to reduce the impact of data imbalance during the training phase.

In the experiments, each model has four blocks. The first one is a convolutional block and the other three are residual blocks. At the end of the model, there are nine classifier blocks for the classification of nine attributes, respectively. We use the reweighted logistic loss to balance the numbers of categories. During the training phase, we set the learning rate to 0.01 with warm restart (29) and use SGD to optimize the model. The momentum was set to 0.09, the weight decay was set to 10^−4^ and the batch size was set to 64. Since the model converges quickly, we have trained 200 epochs for each model and choose the model with the smallest validation loss as the best model.

The imbalanced data causes that no valid features can be learned, and results in low sensitivity of the model to this attribute. As shown in Tables 3, 4, categories with too few samples, such as *partial calcification* and *with cavity*, were not recognized. A given category prediction may have the following four cases: TP, True Positive; FP, False Positive; TN, True Negative; FN, False Negative.

**Table 3:** Performance of the basic model on the 3D, 2.5D, and 2D modes.

**Attributes**	**Categories**	**Accuracy**			**Sensitivity**			**Specificity**		
		**3D**	**2.5D**	**2D**	**3D**	**2.5D**	**2D**	**3D**	**2.5D**	**2D**
Lesion type	Placeholder	0.8636	0.8182	**0.8864**	0.7451	**0.8780**	0.7959	0.9500	0.9344	**0.9561**
	Nodule	0.7460	0.6984	**0.7619**	0.8545	0.8462	**0.9057**	0.8621	0.8288	**0.8636**
	Ground glass opacity	**0.8800**	0.8000	0.8000	**0.8462**	0.8333	0.8000	**0.9793**	0.9640	0.9638
	Air containing space	0.9375	**1.0000**	0.9091	**0.9375**	0.4400	0.7143	0.9935	**1.0000**	0.9933
	Mutation	0.8235	**0.9286**	0.8571	0.8235	**0.8667**	0.8571	0.9805	**0.9932**	0.9866
	Pleural effusion	1.0000	1.0000	1.0000	**1.0000**	**1.0000**	0.7500	1.0000	1.0000	1.0000
Margin	Clear	0.8437	0.8523	**0.8636**	**0.8617**	0.8427	0.8261	0.8052	0.8243	**0.8310**
	Unclear	**0.8267**	0.8133	0.7867	0.8052	0.8243	**0.8310**	**0.8617**	0.8427	0.8261
Spiculation	None	**0.8672**	0.8083	0.8083	0.9407	**0.9604**	**0.9604**	**0.6792**	0.6290	0.6290
	Long	0.3333	0.6000	**0.7333**	**0.4167**	0.2571	0.2558	0.9371	0.9531	**0.9667**
	Short	**0.7143**	0.2857	0.4286	0.4878	0.2963	**0.6316**	**0.9385**	0.8529	0.8889
Lobulation	Without	0.8972	0.8990	**0.9091**	0.9143	0.9271	**0.9375**	0.8333	0.8507	**0.8657**
	With	0.8594	0.8906	**0.9062**	0.8333	0.8507	**0.8657**	0.9143	0.9271	**0.9375**
Calcification	None	**0.9937**	0.8684	0.7763	0.9464	**0.9565**	0.9516	**0.6667**	0.2000	0.1282
	Partial	0.0000	**0.2500**	**0.2500**	0.0000	**0.1176**	0.0741	0.9529	**0.9589**	0.9559
	Total	0.6667	**1.0000**	**1.0000**	**1.0000**	0.3750	0.2500	0.9941	**1.0000**	**1.0000**
Cavity	Without	**0.9819**	0.9557	0.9367	0.9702	0.9742	**0.9801**	0.0000	0.1250	**0.1667**
	With	0.0000	0.2000	**0.4000**	0.0000	0.1250	**0.1667**	0.9702	0.9742	**0.9801**
Vessel convergence	Without	**0.9618**	0.8618	0.8780	0.8936	0.8548	**0.9076**	**0.8333**	0.5641	0.6591
	With	0.6250	0.5500	**0.7250**	**0.8333**	0.5641	0.6591	0.8936	0.8548	**0.9076**
Pleural indentation	Without	**0.8500**	0.8125	0.7946	0.8430	0.8922	**0.9082**	0.6400	**0.6557**	0.6462
	With	0.6275	0.7843	**0.8235**	0.6400	**0.6557**	0.6462	0.8430	0.8922	**0.9082**
Lesion location	Right upper	**1.0000**	0.7083	0.7083	1.0000	1.0000	1.0000	**1.0000**	0.8793	0.8793
	Right middle	**0.8571**	0.7500	0.7500	**1.0000**	0.3000	0.3000	0.9934	**0.9929**	**0.9929**
	Right lower	**1.0000**	0.8750	0.8333	**0.9630**	0.6562	0.7143	**1.0000**	0.9746	0.9672
	Left upper	**1.0000**	0.7143	0.8571	0.9118	**0.9524**	0.9231	**1.0000**	0.9380	0.9677
	Left lower	0.9348	**0.9783**	0.9565	**1.0000**	0.8491	0.8462	0.9739	**0.9897**	0.9796
	Average	0.7513	0.7511	**0.7816**	**0.7671**	0.7006	0.7184	**0.8305**	0.7995	0.8116

*Bold value means better performance, compared between 2D, 2.5D, 3D*.

**Table 4:** Performance of the basic, soft-attention, and self-attention models on the 2D mode.

**Attributes**	**Categories**	**Accuracy**			**Sensitivity**			**Specificity**		
		**Basic model**	**Soft-att**	**Self-att**	**Basic model**	**Soft-att**	**Self-att**	**Basic model**	**Soft-att**	**Self-att**
attention Lesion type	Placeholder	**0.8864**	0.8182	0.8636	**0.7959**	0.7347	0.7755	**0.9561**	0.9298	0.9474
	Nodule	**0.7619**	0.6825	0.7302	0.9057	0.8776	**0.9787**	**0.8636**	0.8246	0.8534
	Ground glass opacity	0.8000	**0.9200**	0.8800	**0.8000**	0.7419	0.7857	0.9638	**0.9848**	0.9778
	Air containing space	0.9091	**1.0000**	0.9091	0.7143	**0.7857**	0.7143	0.9933	**1.0000**	0.9933
	Mutation	0.8571	0.9286	**1.0000**	0.8571	**1.0000**	0.7368	0.9866	0.9933	**1.0000**
	Pleural effusion	**1.0000**	**1.0000**	0.8333	0.7500	**0.8571**	0.8333	**1.0000**	**1.0000**	0.9936
Margin	Clear	**0.8636**	0.8523	**0.8636**	0.8261	**0.8621**	0.8352	0.8310	0.8289	**0.8333**
	Unclear	0.7867	**0.8400**	0.8000	0.8310	0.8289	**0.8333**	0.8261	**0.8621**	0.8352
Spiculation	None	**0.8083**	0.7750	0.7917	0.9604	**0.9894**	0.9500	**0.6290**	0.6087	0.6032
	Long	**0.7333**	0.5333	0.4667	0.2558	**0.3478**	0.2500	**0.9667**	0.9500	0.9407
	Short	0.4286	**0.7500**	0.5357	**0.6316**	0.4565	0.4286	0.8889	**0.9402**	0.8984
Lobulation	Without	0.9091	0.9091	**0.9192**	0.9375	0.9474	**0.9479**	0.8657	0.8676	**0.8806**
	With	0.9062	**0.9219**	**0.9219**	0.8657	0.8676	**0.8806**	0.9375	0.9474	**0.9479**
Calcification	None	0.7763	0.8158	**0.8224**	0.9516	**0.9538**	0.9398	0.1282	**0.1515**	0.1000
	Partial	**0.2500**	**0.2500**	0.0000	0.0741	**0.1111**	0.0000	0.9559	**0.9586**	0.9437
	Total	1.0000	1.0000	1.0000	0.2500	0.2000	**0.3333**	1.0000	1.0000	1.0000
Cavity	Without	0.9367	0.8734	**0.9557**	0.9801	**0.9857**	0.9805	0.1667	0.1304	**0.2222**
	With	0.4000	**0.6000**	0.4000	0.1667	0.1304	**0.2222**	0.9801	**0.9857**	0.9805
Vessel convergence	Without	0.8780	0.7967	**0.8211**	0.9076	**0.9515**	0.9182	**0.6591**	0.5833	0.5849
	With	0.7250	**0.8750**	0.7750	**0.6591**	0.5833	0.5849	0.9076	**0.9515**	0.9182
Pleural indentation	Without	**0.7946**	0.7857	0.7589	0.9082	0.9167	**0.9551**	**0.6462**	0.6418	0.6351
	With	0.8235	0.8431	**0.9216**	**0.6462**	0.6418	0.6351	0.9082	0.9167	**0.9551**
lesion Location	Right upper	0.7083	0.7083	0.7083	**1.0000**	**1.0000**	0.9714	**0.8793**	**0.8793**	0.8783
	Right middle	0.7500	0.7500	0.7500	0.3000	**0.3333**	0.3000	0.9929	0.9929	0.9929
	Right lower	0.8333	0.8333	**0.8750**	0.7143	0.6897	**0.7241**	0.9672	0.9669	**0.9752**
	Left upper	0.8571	0.8214	**0.9286**	0.9231	0.9200	**0.9630**	0.9677	0.9600	**0.9837**
	Left lower	0.9565	0.9565	0.9565	0.8462	0.8302	**0.8980**	0.9796	0.9794	**0.9802**
	Average	0.7816	**0.8032**	0.7763	**0.7184**	0.7183	0.7155	0.8116	0.8117	**0.8128**

*Bold value means better performance, compared between different models*.

To evaluate the imbalanced categories of each attribute, we use three metrics to score the results. **Accuracy** (ACC) is the basic metric to evaluate the result, which can be calculated as:


(5) $$\begin{array}{l} ACC=\frac{TP+TN}{P+N} \end{array}$$

**Sensitivity** (SE), also called the true positive rate, means the probability that a sick person is diagnosed as positive, which can be calculated as:


(6) $$\begin{array}{l} SE=\frac{TP}{TP+FN} \end{array}$$

The larger the SE value, the more sensitive our model is in diagnosing this category.

**Specificity** (SP), also called the true negative rate, means the probability that a person who is actually not sick is diagnosed as negative, which can be calculated as:


(7) $$\begin{array}{l} SP=\frac{TN}{FP+TN} \end{array}$$

The larger the value of SP, the more accurate our model is for the diagnosis of this category.

We average out accuracies of all categories for each attribute, and average the scores of all attributes as the final score to represent the performance of the model.

### 6.1. Results for Input Modes

**Figure 5:**
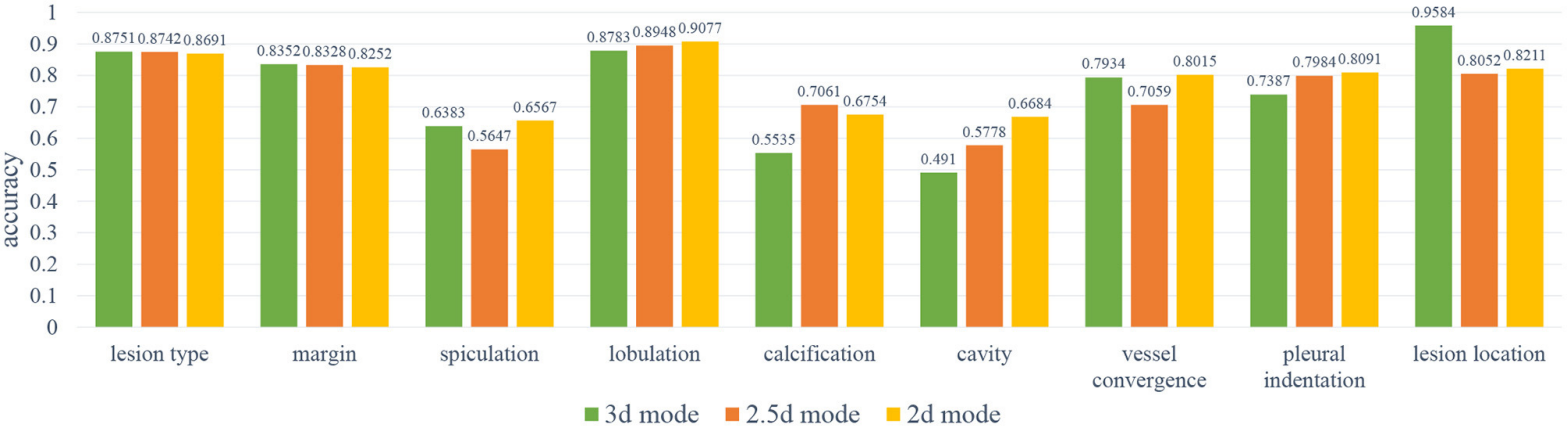
The results of the 2D, 2.5D, 3D input modes. As can be noted, the 3D mode has better results on spiculation, lobulation, cavity, vessel convergence, and pleural indentation.

In order to select the most suitable input mode for the attribute classification of lung lesions, we train the 2D, 2.5D, and 3D model with the same structure described in Figure 3. To ensure the fairness of the three models, we do not adjust the hyper-parameters for different models. Each model was trained with 200 epochs and a batch size of 64. To evaluate the performance of the models, we chose the average accuracy of the model with the lowest validation loss as the metric. The average accuracy scores of the 3D, 2.5D, 2D model are 0.7513, 0.7511, and 0.7816; the average sensitivity are 0.7671, 0.7006, and 0.7184; and the average specificity are 0.8305, 0.7995, and 0.8116, respectively. As Figure 5 shows, the three models have almost the same scores in lesion type and margin, and the model with 2D mode has better scores in spiculation, lobulation, vessel convergence, and pleural indentation. Table 3 shows the accuracy, sensitivity, and specificity of each category for each attribute. From the experimental results, we note that the higher-level attributes, such as lesion type and lesion location, are more sensitive to the 3D mode and the lower-level attributes, such as spiculation and lobulation, are more sensitive to the 2D mode.

During training, we noticed that the 3D model has more parameters than the 2D models, which led to longer training time and slower convergence. Meanwhile, the 2D model has better average accuracy than the 3D model. So, we chose the 2D mode as the basic model for the following experiments.

### 6.2. Results for Attention Mechanisms

In order to improve the performance of the basic model, we have used two attention mechanisms to enhance the feature before feeding it to the classifiers. We called the model with the soft-attention module *Soft-Att*, and the model with the self-attention module *Self-Att*. Since the number of parameters of the two attention modules is not large, we use the same hyper-parameters as the basic model to train the two models. Similar to the previous section, we used a batch size of 64 and 200 epochs for training and taking the accuracy of the model with the lowest validation loss as the metric. The average accuracies scores of the basic model, Soft-Att and Self-Att are 0.7816, 0.8032, and 0.7763; the average sensitivities are 0.7184, 0.7183, and 0.7155; and the average specificities are 0.8116, 0.8117, and 0.8128, respectively.

**Figure 6:**
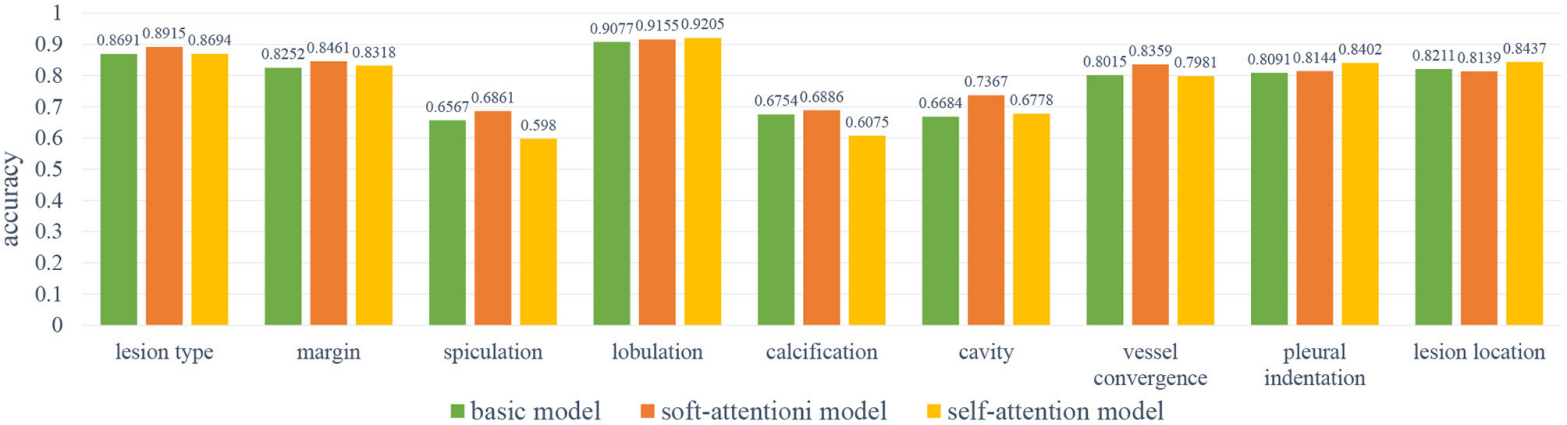
The results of the base model and two attention models. As can be noted, the self-attention module has better results on lobulation, pleural indentation, and lesion location attributes; the soft-attention module has better results on lesion type, margin, spiculation, calcification, cavity, and vessel convergence attributes.

As Figure 6 shows, the soft-attention module has better results on margin, vessel convergence, lesion type, and spiculation attributes, and the self-attention module has better results on lobulation, pleural indentation, and lesion location attributes. Due to the near-zero sensitivity of calcification and cavity attributes, we do not take their accuracy into comparison. As reported in Table 4, the two models with attention modules have better performance than the basic model.

**Figure 7:**
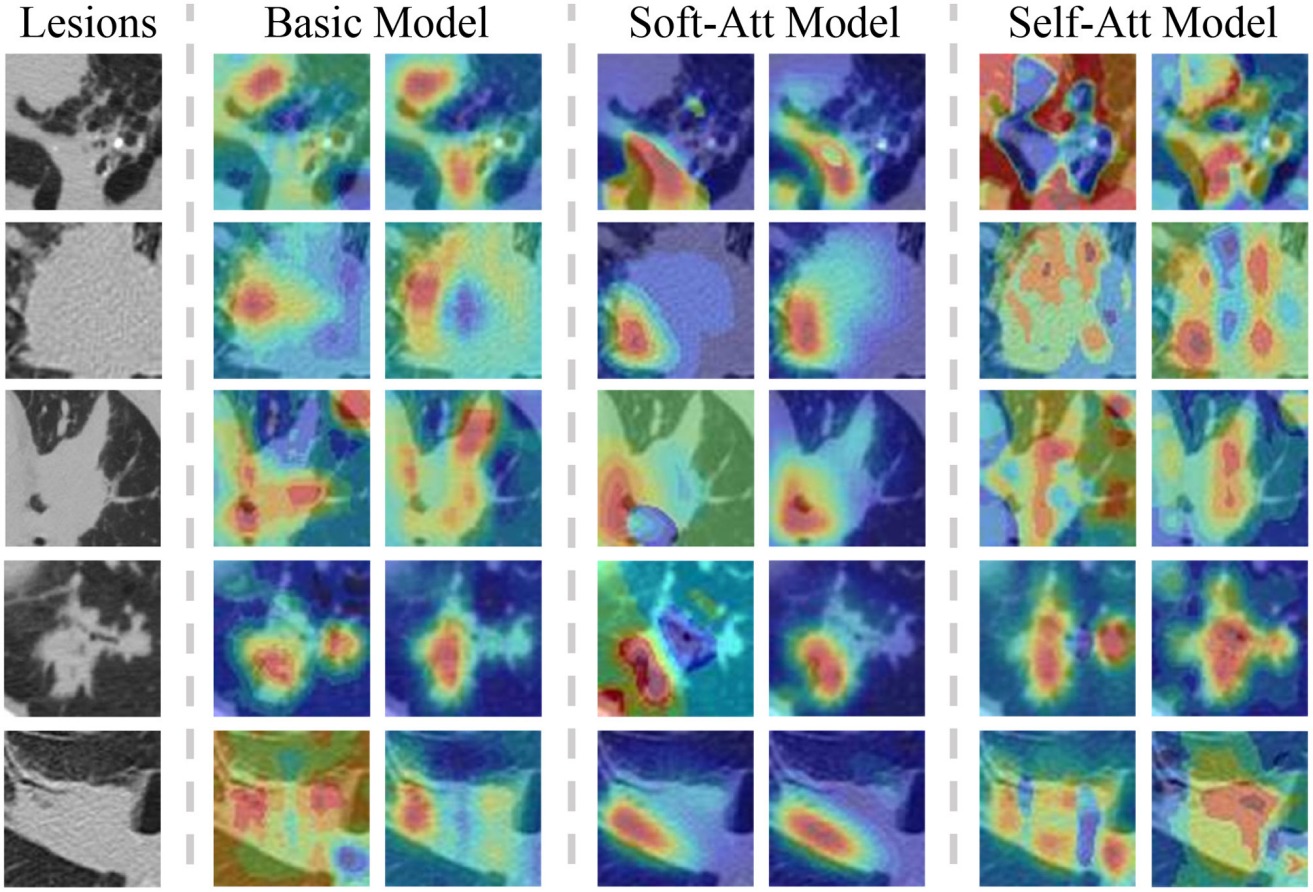
The lesions with two heatmaps from the basic model, soft-Att, self-Att, respectively, in the lesion type and lobulation attributes. The color means the importance of the feature in that position. The red color indicates an important feature. As the figures show, the attention model focuses the features on one point and self-attention spreads the features in the spatial dimension.

The heatmaps in Figure 7 visualize the attention mechanisms. Compared with the basic model, the red value of soft-attention is concentrated at one point. This is because soft-attention uses higher-layer semantic information to filter the low-layer features, which makes the features spatially smoother and more focused. This is a good feature for high-level attributes because it is concentrated at the point that best reflects the attribute, but it does not fully reflect the local information relationship. Compared with the basic model, the red value of self-attention is more scattered in the spatial dimension. This is because self-attention extracts channel information by compressing spatial information using its own features, and it is more comprehensive in spatial information due to multi-channel fusion. This is a good feature for low-level attributes because its local information relationships are more spatially refined, but because of the noise in the spatial dimension, it may not be appropriate for high-level attributes.

## 7. Conclusion

This paper presents a dataset of lung lesions with fine contour annotation and attribute and explores the correlation between the attributes of the dataset. To demonstrate the contribution of this dataset to the development of CAD systems, we explore two issues of medical data modeling using attribute classification tasks.

One of the issues is the effect of the 2D, 2.5D, 3D input mode on the classification model. The 2D mode works well for low-level attributes that do not require local information relationships between lesions and surrounding tissues, while the 3D mode works better for high-level attributes that require higher contextual relationships. The 2.5D mode is a trade-off between the lightweight of the 2D model and the context information of the 3D model.

The second is the impact of the two attention mechanisms on the model. Soft-attention can better handle the noise in the spatial dimension and concentrate on the features at one point, which is beneficial for the classification of high-level attributes. Self-attention can better integrate the spatial information in the channel dimension, and complement the local relationship in the spatial dimension, which is beneficial for the classification of low-level attributes.

In the future, we mainly want to explore and address the following three issues:

1. For the three categories of cavity, partial calcification, and long spiculation, the sensitivity is almost zero due to the high degree of the category imbalance. We will explore novel methods to improve the accuracy of these three categories.
2. We will use the correlation between attributes to establish a loss function suitable for multi-attribute classification from the statistical learning strategy.
3. There is not a single metric that can well measure the performance of a multi-attribute model. We will build evaluation metrics for multi-task modeling.

## Data Availability Statement

The original contributions presented in the study are included in the article/supplementary material, further inquiries can be directed to the corresponding author/s.

## Author Contributions

All authors listed have made a substantial, direct and intellectual contribution to the work, and approved it for publication.

## Conflict of Interest

The authors declare that the research was conducted in the absence of any commercial or financial relationships that could be construed as a potential conflict of interest.
